# Genomic risk for post-traumatic stress disorder in families densely affected with alcohol use disorders

**DOI:** 10.1038/s41380-023-02117-9

**Published:** 2023-06-21

**Authors:** Stacey Saenz de Viteri, Jian Zhang, Emma C. Johnson, Peter B. Barr, Howard J. Edenberg, Victor M. Hesselbrock, John I. Nurnberger, Ashwini K. Pandey, Chella Kamarajan, Sivan Kinreich, Jay A. Tischfield, Martin H. Plawecki, John R. Kramer, Dongbing Lai, Samuel Kuperman, Grace Chan, Vivia V. McCutcheon, Kathleen K. Bucholz, Bernice Porjesz, Jacquelyn L. Meyers

**Affiliations:** 1 State University of New York Downstate Health Sciences University, Brooklyn, NY USA; 2 Washington University School of Medicine in St. Louis, St. Louis, MO USA; 3 Indiana University School of Medicine, Indianapolis, IN USA; 4 University of Connecticut Health Center, Farmington, CT USA; 5 Rutgers University, Piscataway, NJ USA; 6 University of Iowa Carver College of Medicine, Iowa City, IA USA

**Keywords:** Genetics, Molecular biology

## Abstract

Recent genome-wide association studies (GWAS) have identified genetic markers of post-traumatic stress disorder (PTSD) in civilian and military populations. However, studies have yet to examine the genetics of PTSD while factoring in risk for alcohol dependence, which commonly co-occur. We examined genome-wide associations for DSM-IV PTSD among 4,978 trauma-exposed participants (31% with alcohol dependence, 50% female, 30% African ancestry) from the Collaborative Study on the Genetics of Alcoholism (COGA). We also examined associations of polygenic risk scores (PRS) derived from the Psychiatric Genomics Consortium (PGC)-PTSD Freeze 2 (*N* = 3533) and Million Veterans Program GWAS of PTSD (*N* = 5200) with PTSD and substance dependence in COGA, and moderating effects of sex and alcohol dependence. 7.3% of COGA participants met criteria for PTSD, with higher rates in females (10.1%) and those with alcohol dependence (12.3%). No independent loci met genome-wide significance in the PTSD meta-analysis of European (EA) and African ancestry (AA) participants. The PGC-PTSD PRS was associated with increased risk for PTSD (*B* = 0.126, *p* < 0.001), alcohol dependence (*B* = 0.231, *p* < 0.001), and cocaine dependence (*B* = 0.086, *p* < 0.01) in EA individuals. A significant interaction was observed, such that EA individuals with alcohol dependence and higher polygenic risk for PTSD were more likely to have PTSD (*B* = 0.090, *p* < 0.01) than those without alcohol dependence. These results further support the importance of examining substance dependence, specifically alcohol dependence, and PTSD together when investigating genetic influence on these disorders.

## Introduction

Post-traumatic stress disorder (PTSD) is a psychiatric disorder that occurs after experiencing a traumatic event [1]. Approximately 50-89% of the United States population is exposed to some type of traumatic event during their lifetime [2, 3]; however, only 6–8% of the population is diagnosed with PTSD [1]. This may be due to different factors that contribute to risk for or resilience to developing PTSD, such as socio-demographic characteristics (e.g., female gender), comorbid mental health problems (e.g., alcohol use disorders), and genetic liability [4–8].

Twin and molecular studies have shown that PTSD is moderately heritable *(h*^*2*^ = 5–34%) [6, 8–10], and research has begun to identify risk loci for PTSD. The Psychiatric Genomics Consortium (PGC)-PTSD Workgroup carried out a Genome-Wide Association Study (GWAS) of PTSD in a multi-ethnic cohort that included over 30,000 PTSD cases and 170,000 controls [9]. The PGC-PTSD workgroup combines genetic information from over 72,000 civilian and military samples to create a large, ancestrally and sex diverse database with approximately 40% of the population being female and 30% of African ancestry [9]. Genome-wide significant variants were identified in those of European (rs345178562, rs9364611) and African (rs115539978) ancestries (EA and AA, respectively), with three additional variants identified in males only (EA males: rs148757321, rs571848662; AA males: rs1421744523). While none of the 6 leading SNPs were replicated in the Million Veteran Program (MVP) cohort, a diverse sample of >165,000 veterans assessed for PTSD with re-experiencing symptoms [10], polygenic risk scores (PRS) derived from the PGC-PTSD study [9] were associated with PTSD re-experiencing symptoms in the MVP [10]. The MVP cohort is also an ancestrally diverse population, with 11% of their PTSD GWAS sample being of African ancestry. However, it is a military and predominantly male sample, with approximately 6% of the GWAS population being female [10]. Further, PTSD PRS derived from the PGC-PTSD study have been associated with PTSD in independent samples [11]. In addition, other studies have used alcohol use-related PRS to predict alcohol and other substance use disorders [12]. However, no study to our knowledge has used PTSD PRS to predict alcohol and other substance use disorders.

Approximately one-third of individuals with PTSD have co-occurring alcohol dependence [5]. In addition, individuals with a family history of alcohol dependence are at increased risk of experiencing a traumatic event and developing PTSD [13–16], potentially due to shared psychosocial risk factors (i.e., adverse childhood experiences) and/or the shared genetic liability of PTSD and alcohol dependence and other substance use disorders [6, 8, 17]. Using GWAS data, Sheerin et al. (2020) confirmed a significant genetic correlation between PTSD and alcohol dependence (rG = 0.35) [8], first observed in twin studies [6, 8, 17–19]. Given sex differences in prevalence of PTSD [4, 20] and sex differences in the heritability of PTSD [9, 21, 22], Sheerin and colleagues conducted sex-stratified analyses, and found a significant genetic correlation between PTSD and alcohol dependence in females (rG = 0.34), but not in males (rG = 0.14) [8].

While recently published GWAS [9, 21, 22] have begun to identify genomic markers of PTSD in civilian and military populations, these studies have yet to examine the genetics of PTSD while considering co-occurring influences of alcohol dependence. Further, whether the shared risk between PTSD and alcohol use disorders extends to other substance use disorders is less clear. While recent studies have suggested that genetic risk is shared across a range of externalizing disorders (including alcohol, cannabis, and nicotine use disorders) [12], prior research has demonstrated that some risk factors may be unique to each substance use disorder [23].

The existing epidemiological literature is mixed with regard to sex differences in the prevalence of comorbid PTSD and alcohol dependence [4, 24–27]. Men are more likely to be exposed to a traumatic event. However, women are more likely to be diagnosed with PTSD [4, 24]. Additionally, men are more likely to be diagnosed with alcohol-related disorders, while women with alcohol dependence are more likely than men to be diagnosed with PTSD [4, 24]. These studies have shown that PTSD and alcohol dependence diagnosis and presentation are different between men and women. While most studies focus on prevalence and diagnosis, few genetically informative studies have examined sex differences in comorbid PTSD, alcohol and other substance dependence diagnoses [28–31]. Additional research is needed to better understand the factors that moderate the relationship between sex and PTSD-AUD comorbidity.

The current study examines genome-wide associations with PTSD in the *Collaborative Study on the Genetics of Alcoholism* (COGA), a study of families many of which are densely affected with alcohol use disorders. Primary analyses will examine the relation of recent GWAS of PTSD [9, 22] to PTSD, alcohol dependence, and other substance dependence diagnoses, including cannabis, cocaine, and opioid dependence in the COGA sample, and how these associations may differ between males and females. Exploratory analyses will examine relations between PTSD PRS and PTSD and other substance dependence diagnoses in individuals with and without alcohol dependence.

## Material and methods

### GWAS sample and measures

The Collaborative Study on the Genetics of Alcoholism (COGA) is a large, multi-site study of 17,854 participants from 2255 families, many of which are densely affected with AUD, designed to identify and understand genetic factors involved in the predisposition to develop alcohol use disorder and related disorders, as previously described [32, 33]. Beginning in 1990, participants were administered a comprehensive battery that included clinical and neurophysiological assessments, and DNA samples for genomic analysis (see [32] for description of recruitment and assessment procedures). Clinical assessments of substance use and psychiatric disorders were obtained using the Semi-Structured Assessment for the Genetics of Alcoholism (SSAGA) developed in COGA, which is a reliable and valid poly-diagnostic interview [34, 35]. A developmentally appropriate version of the SSAGA was administered to those under age 18 [36]. Starting in 1997, assessments for trauma exposure were added to the SSAGA; participants who reported a qualifying trauma were then queried about symptoms of PTSD that were required for a DSM-IV diagnosis (Diagnostic and Statistical Manual of Mental Disorders-IV) [35, 37]. For the purpose of this study, data were included only for COGA participants that were assessed for trauma exposure and/or PTSD. Of the 17,854 COGA participants, 8878 were assessed for lifetime DSM-IV PTSD diagnosis. Because exposure to trauma is needed for a PTSD diagnosis, and to remain consistent with published GWAS, the analytic sample for our study was limited to the 4978 out of 8878 (56%) participants assessed for DSM-IV PTSD (AA *N* = 1468; EA *N* = 3510) who were both genotyped and reported experiencing a DSM-based qualifying traumatic event. This was done so as to not conflate genetic influences on trauma exposure and genetic influences on PTSD. Although the statistical power is relatively limited, especially for the AA sample, we conducted this analysis to emphasize the importance of including underrepresented populations in genetic studies. Descriptive statistics for the GWAS analytic sample are displayed in Supplemental Table 1.

### Genotyping, imputation and quality control

As described previously [38], genotyping was performed using the Illumina 2.5 M array (Illumina, San Diego, CA, USA), the Illumina OmniExpress [39], the Illumina 1 M array, or the Affymetrix Smokescreen array [40]. Single nucleotide polymorphisms (SNPs) with a genotyping rate <98%, or that violated Hardy-Weinberg equilibrium (*p* < 10^−6^), or with minor allele frequency (MAF) less than 3% were excluded from analyses. Mendelian inconsistencies were removed, after which data were imputed to 1000 genomes (EUR and AFR, Phase 3, b37, October 2014; build hg19) using SHAPEIT [41] and IMPUTE2 [42]. Following imputation, genotype probabilities ≥0.90 were changed to genotypes. Mendelian errors in the imputed SNPs were reviewed and resolved as described previously [43, 44]. SNPs with an imputation information score <0.30 or MAF <0.03 were excluded from subsequent analysis.

### GWAS statistical analysis

Genetic analysis was conducted on 7,009,929 SNPs in the European ancestry (EA) sample and 13,862,444 SNPs in the African ancestry sample by the generalized disequilibrium test (GDT) method [45] using family-based information. Principal components (PCs) derived from GWAS data were used to assign ancestry in the genotyped sample, and families were classified as EA or AA according to the ancestry of the greatest proportion of family members. Analyses were conducted separately in the families of AA and EA, using identical phenotypic definitions, covariates, SNP QC standards, MAF thresholds and imputation protocols. Sex, age, the first three PCs (PC1-PC3) computed from SNPRelate, and genotype array were included as covariates. Subsequently, meta-analysis across the EA and AA samples was performed using inverse-variance weighting and genomic control in METAL [46]. Established thresholds for genome-wide significance (*p* < 5 × 10^−8^) were used. We performed MAGMA [47] competitive gene-set analyses using the summary statistics from the GWAS of PTSD in EA and AA individuals using FUMA v1.3.6a (Functional Mapping and Annotation of Genome-Wide Association Studies) [48]. The Genotype-Tissue Expression (GTEx v8 [49]) database was used to obtain gene expression levels in 10 different brain regions.

### Polygenic Risk Score (PRS) sample, measures, and statistical analyses

Data used to calculate the polygenic risk score (PRS) from the PGC-PTSD Freeze 2 summary statistics were available for only EA participants (*N* = 3533 trauma-exposed individuals). In contrast, both EA and AA participants comprised the MVP-PTSD PRS (EA = 3522 and AA = 1678 trauma-exposed individuals). Descriptive statistics for the PGC-PTSD PRS and MVP-PTSD PRS analytic subsamples are displayed in Supplementary Tables 2 and 3, respectively. Additional measures used in both PRS analyses included lifetime DSM-IV alcohol dependence (endorsement of 3 or more out of 7 symptoms).

PRS were generated from the PGC-PTSD Freeze 2 data [9] for European ancestry (EA) COGA participants (*N* = 3533) using PRS-cs [50]. We tested the association of the PGC-PTSD PRS with lifetime DSM-IV PTSD and substance (alcohol, cannabis, cocaine, opioid) dependence diagnoses, including sex (female = 1; male = 0), age, ancestral principal components, and genotype array as covariates in EA COGA participants. Next, exploratory analyses were performed to investigate interaction effects of lifetime alcohol dependence with PRS (i.e., PRS*sex, PRS*alcohol dependence diagnosis) given that alcohol dependence has been shown to increase the likelihood of being diagnosed with PTSD and other (non-alcohol) substance dependence [5]. Lifetime PTSD diagnosis and non-alcohol substance (cannabis, cocaine, opioid) dependence diagnoses were included simultaneously as outcomes in the pathway model to account for the correlation between these outcomes. All association analyses were conducted in MPlus [51], clustering for familial relatedness, and included cross-term interactions as covariates (e.g., PRS*sex, PRS*age, etc.), as these have been shown to potentially bias effects in analyses involving PRS [52]. In addition, including cross-term interactions allowed for the primary analysis to investigate PRS*sex interaction effects. All analyses were repeated using the Million Veteran Program (MVP) PRS that were generated from summary statistics for the MVP GWAS on DSM-IV PTSD diagnosis [22] for European (EA) and African ancestry (AA) COGA participants (N: EA = 3522; AA = 1678) using PRS-csx [22]. To account for multiple comparisons while taking into consideration the correlation between our outcome variables (i.e., PTSD and substance dependence diagnoses; Supplementary Tables 4 and 5), we calculated adjusted *p*-values using the Benjamini–Hochberg Procedure [53] to decrease the false discovery rate in our models. Given that COGA’s sample is densely affected with alcohol use disorders, secondary exploratory analyses also included a problematic alcohol use PRS calculated from Barr et al. (2022) as a covariate to investigate any main and interaction effects of polygenic risk for problematic alcohol use in our sample [54].

## Results

### GWAS of PTSD

Of the trauma-exposed sample 7.3% met criteria for PTSD, with higher rates observed in females (10.5%) than in males (4.2%), and similar rates in EA (7.3%) and AA (7.3%) participants.

No individual loci met the genome-wide significance criteria (*p* < 5 × 10^−8^) in the meta-analysis of PTSD (Supplementary Fig. 1). The 10 top SNPs, representing five independent loci, are listed in Supplementary Table 6. The lead SNP for each locus included: Follistatin Like 4 (*FSTL4)* upstream variant SNP rs2457174, located on chromosome 5 (*p* = 2.9 × 10^−7^), intronic Fibronectin Type III And Spry Domain Containing 1 Like (*FSD1L)* SNP rs9969773 on chromosome 9 (*p* = 4.0 × 10^−7^), Cillia And Flagella Associated Protein 54 (*CFAP54)* downstream variant SNP rs71439002 on chromosome 12 (*p* = 9.8 × 10^−7^), intergenic chromosome 4 SNP rs7668166 (*p* = 1.1 × 10^−6^), and intergenic chromosome 5 SNP rs67697853 (*p* = 1.4 × 10^−6^).

### PGC-PTSD PRS in COGA participants of European Ancestry

**Fig. 1:**
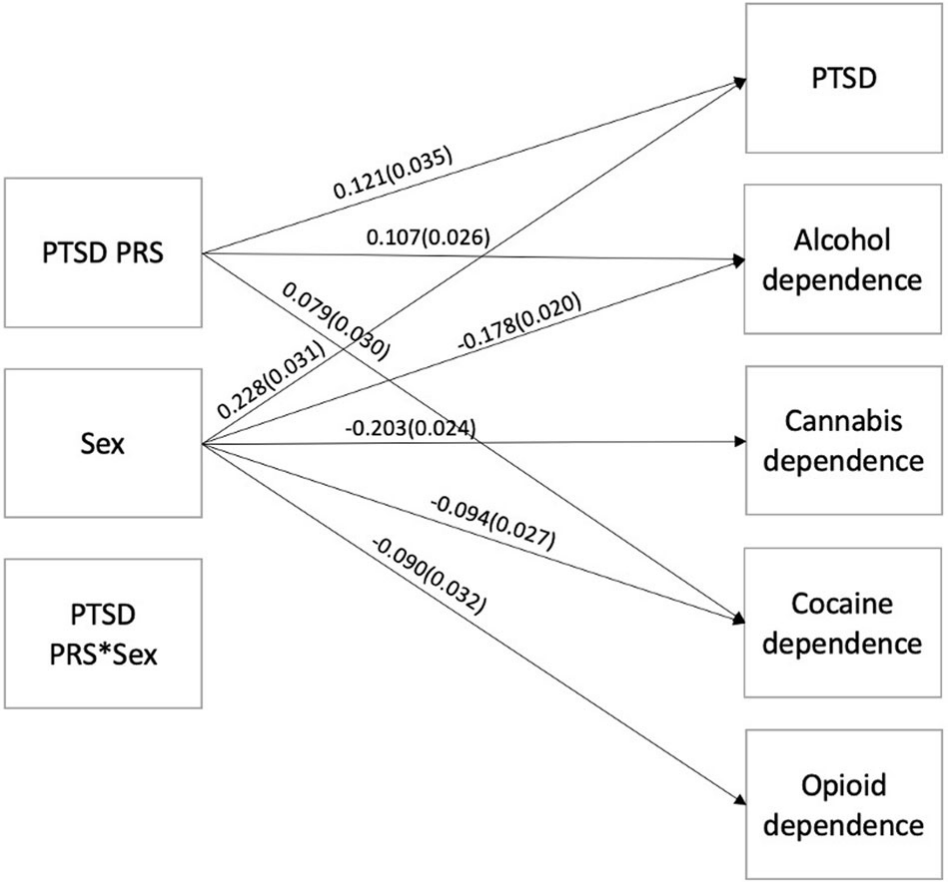
Main and interaction effects of PGC-PTSD PRS and sex on DSM-IV PTSD and substance dependence in COGA. Note: Parameter estimates (and standard errors) are displayed only for statistically significant pathways that remained significant after adjusting *p*-values for multiple testing using the Benjamini–Hochberg procedure. Not pictured, but also included in this model as covariates, are age, principal components, genotype array, and cross-term covariate interactions.

**Fig. 2:**
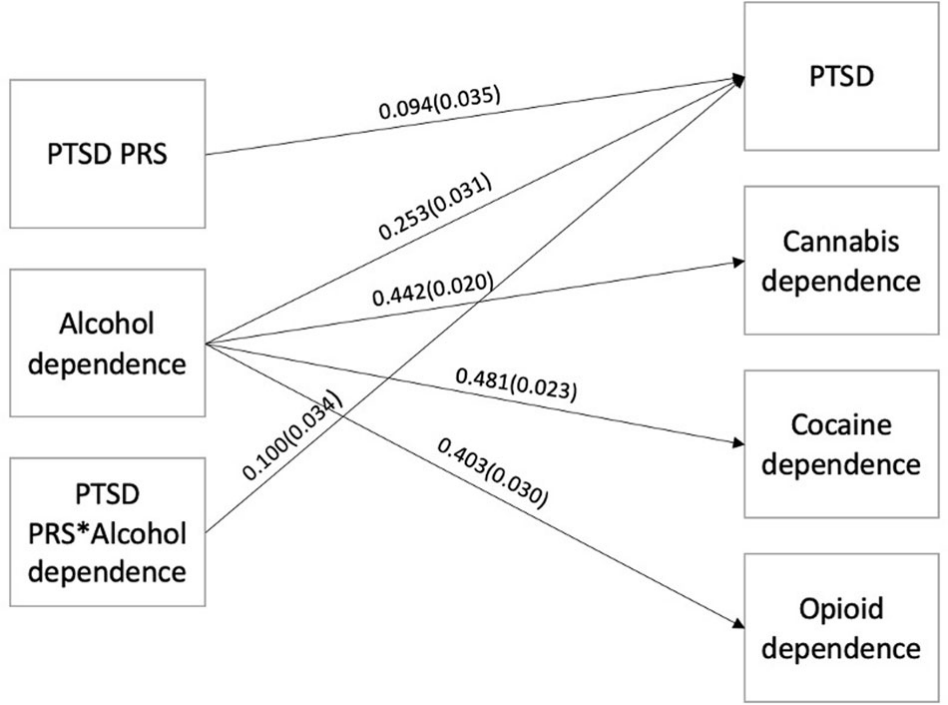
Associations between PGC-PTSD PRS*Alcohol dependence and DSM-IV PTSD and substance dependence in COGA. Note: Parameter estimates (and standard errors) are displayed only for statistically significant pathways that remained significant after adjusting *p*-values for multiple testing using the Benjamini–Hochberg procedure. Not pictured, but also included in this model as covariates, are sex, age, principal components, genotype array, and cross-term covariate interactions.

In the COGA sub-sample used for the PGC-PTSD PRS analyses, 7.3% of the sample met criteria for PTSD (Supplementary Table 2). The highest prevalent substance dependence diagnosis was alcohol dependence (32.7%), followed by cannabis dependence (20.4%), cocaine dependence (10.2%), and opioid dependence (5.9%). The PGC-PTSD PRS [9] was associated with increased risk for DSM-IV diagnoses of PTSD (*B* = 0.121, *p* < 0.01, adjusted-*p* < 0.01; R2change = 0.013), as well as DSM-IV alcohol- (*B* = 0.107, *p* < 0.001, adjusted-*p* < 0.001; R2change = 0.011) cannabis- (*B* = 0.063, *p* < 0.05, adjusted-*p* > 0.05; R2change = 0.003) and cocaine- (*B* = 0.079, *p* < 0.01, adjusted-*p* < 0.05; R2change = 0.006) dependence, but not with opioid dependence (Supplementary Table 7; Fig. 1). Addition of the problematic alcohol use PRS changed the significance for the cocaine finding, but not the other findings, such that it was no longer significant (Supplementary Table 8). Significant main effects for sex were observed for PTSD and all substance dependence diagnoses, such that female participants were more likely to be diagnosed with PTSD (*B* = 0.228, *p* < 0.001, adjusted-*p* < 0.001) and less likely to be diagnosed with alcohol (*B* = −0.178, *p* < 0.001, adjusted-*p* < 0.001), cannabis (*B* = −0.203, *p* < 0.001, adjusted-*p* < 0.001), cocaine (*B* = −0.094, *p* < 0.001, adjusted-*p* < 0.001), or opioid (*B* = −0.090, *p* < 0.01, adjusted-*p* < 0.05) dependence, compared to male participants (Supplemental Table 7; Fig. 1). The significant associations between the PGC-PTSD PRS and PTSD, alcohol dependence and cocaine dependence, but not cannabis dependence, remained significant after adjusting p-values for multiple testing using the Benjamini–Hochberg procedure, as did the significant main effects observed for sex. No significant interaction effects of sex on the associations between the PGC-PTSD PRS and PTSD and substance dependence diagnoses were observed (Supplementary Table 7; Fig. 1). Interaction effects of alcohol dependence were observed, such that individuals with a lifetime alcohol dependence diagnosis and higher polygenic risk for PTSD were more likely to have PTSD (*B* = 0.100, *p* < 0.01, adjusted-*p* < 0.05) compared to individuals with higher polygenic risk for PTSD, but without alcohol dependence (Supplementary Table 9; Fig. 2). This finding remained significant after adjusting p-values for multiple testing using the Benjamini–Hochberg procedure. Further, the addition of the problematic alcohol use PRS changed the main effect of the PGC-PTSD PRS on PTSD diagnosis. While the association was still nominally significant (*p* < 0.05), it was no longer significant with the Benjamini–Hochberg procedure. However, the inclusion of the problematic alcohol use PRSS in the model did not change the significant interaction effect between the PGC-PTSD PRS and alcohol dependence (Supplementary Table 10).

### MVP-PTSD PRS in COGA participants of European (EA) and African (AA) Ancestry

In the COGA sub-sample used for MVP-PTSD PRS analyses, 7.4% of the population met criteria for PTSD (Supplementary Table 3). The highest prevalent substance dependence diagnosis was alcohol dependence (29.6%), followed by cannabis dependence (22.0%), cocaine dependence (10.6%), and opioid dependence (5.0%). Of the EA sub-sample, 7.4% met criteria for PTSD, 32.8% for alcohol dependence, 20.5% for cannabis dependence, 10.3% for cocaine dependence, and 5.9% for opioid dependence. In the AA sub-sample, 7.3% met criteria for PTSD, while the highest prevalent substance dependence diagnosis was cannabis dependence (25.3%), followed by alcohol dependence (22.8%), cocaine dependence (11.3%), and opioid dependence (3.2%). No significant main effects of the MVP-PTSD PRS were observed for DSM-IV diagnoses in EA or AA individuals (Supplementary Table 11). In addition, no significant interaction effects (i.e., PRS*sex or PRS*alcohol dependence) were observed (Supplementary Tables 11 and 13, respectively). Further, the addition of the problematic alcohol use PRS did not change any of these findings (Supplementary Tables 12 and 14).

## Discussion

This GWAS of PTSD in COGA families, many of which are densely affected with alcohol use disorders, yielded no genome-wide significant loci. However, associations of polygenic risk for PTSD were observed with DSM-IV PTSD as well as alcohol and cocaine dependence using PRS calculated from PGC-PTSD Freeze 2 data [9]. Further, individuals with increased polygenic risk for PTSD and a lifetime alcohol or cocaine dependence diagnosis were more likely to have PTSD than those without alcohol or cocaine dependence. The main effects observed for the PGC-PTSD PRS were not seen when investigating the same associations using PRS calculated from the MVP GWAS on PTSD diagnosis.

PTSD and substance use problems, such as alcohol and cocaine dependence, are complex disorders that commonly co-occur [5, 55]. However, gaps in knowledge exist regarding the reasons for this link between PTSD and substance use disorders. One theory, the shared-liability model, suggests that individuals with co-occurring PTSD and substance use problems have shared risk factors, such as genetic risk [6, 8]. While studies have shown significant genetic overlap (rG = 0.35) between PTSD and alcohol dependence [8], it is important to determine potential shared risk factors that may further link these two disorders. Few studies have investigated associations between PTSD-PRS and other commonly co-occurring disorders [56]. This is the first study to our knowledge that has shown associations between polygenic risk of PTSD and alcohol and cocaine dependence in a sample densely affected with alcohol use disorders. The PGC-PTSD PRS accounted for 1.3%, 1.1%, and 0.6% of the variability in PTSD, alcohol dependence and cocaine dependence, respectively. While effect sizes are quite modest, these results extend previous research that have shown significant genetic correlations between PTSD and alcohol dependence [6, 8], further suggesting that there is shared genetic risk for these disorders. These results further support the importance of exploring the co-occurrence of substance use problems, specifically alcohol dependence and PTSD, when investigating genetic influence on either of these disorders.

The current study showed significant main effects of sex on PTSD and substance dependence diagnoses that are consistent with the existing literature [5, 24]. However, no significant interaction was seen between sex and the PGC-PTSD PRS on PTSD or substance dependence diagnoses, suggesting that sex does not play a moderating role in the relationship between polygenic risk of PTSD and PTSD or substance dependence. Given that men and women are known to be exposed to different types of trauma [24] and the current study was limited to investigating any trauma exposure, future studies should investigate whether trauma type may interact with polygenic risk of PTSD in men and women to observe any potential differences.

The MVP PTSD PRS did not predict PTSD diagnosis among either EA or AA COGA sub-samples. It is possible that this is a result of different sample composition for the MVP and COGA. For example, the MVP sample is predominantly male and consists of military veterans [22], whereas the COGA sample has closer to equal numbers of male and female participants and is a civilian population. While the MVP is a large and ancestrally diverse GWAS, it is possible that the relatively smaller AA samples in both the MVP and COGA study, could affect the MVP PTSD-PRS associations in the COGA sample. Clearly, more ancestrally diverse GWAS are needed to understand and replicate these findings.

### Limitations

While these results provide important information regarding the relationship between PTSD and alcohol dependence, there were limitations that should be considered. The present study was small in comparison to other GWAS. Larger sample sizes are desirable for genetic association studies due to the small effects of loci typically detected in GWAS. Further, these analyses do not consider the temporality of PTSD and alcohol dependence diagnoses. Future studies should examine the age or timing of onset for each disorder to unpack the causal relationships between PTSD and alcohol dependence beyond shared genetic risk. In addition, limited information was available on trauma exposure type (e.g., assaultive, nonassaultive) for the entire sample. Initial phases in COGA only assessed whether an individual experienced a traumatic event without asking the individual specific questions regarding their traumatic exposure. Therefore, influence of trauma type on these associations was not investigated. Given the known sex differences in exposure rates and influences of different trauma types on risk of PTSD and alcohol dependence, future studies should examine whether polygenic risk of PTSD differs depending on trauma type.

In addition, the current study investigated associations between PTSD PRS, PTSD, and different substance dependence diagnoses simultaneously. While this statistical model allowed the diagnoses to covary, it is not a test of comorbidity (i.e., the model does not predict the relationship between PTSD PRS and co-morbid diagnoses). Since studies, including the current study, have demonstrated the importance of investigating PTSD and substance use disorders together, future studies with larger sample sizes should investigate genetic risk of comorbid PTSD and substance dependence, especially alcohol dependence.

## Conclusions

In conclusion, polygenic risk of PTSD, derived from the PGC-PTSD most recent GWAS on PTSD diagnosis, was associated with increased likelihood of DSM-IV PTSD, and DSM-IV alcohol and cocaine dependence diagnoses in an independent sample of individuals densely affected with alcohol use disorders. Further, individuals with higher polygenic risk for PTSD and a lifetime alcohol dependence diagnosis were more likely to have PTSD than those without alcohol dependence. These results further support the importance of examining substance use problems, specifically alcohol dependence, and PTSD together when investigating genetic influence on these disorders.

### Supplementary information

Supplemental Material
