# Supplementary material for: Genomic risk for post-traumatic stress disorder in families densely affected with alcohol use disorders
Source: Mol Psychiatry. 2023 Jun 21;28(8):3391–6. doi: 10.1038/s41380-023-02117-9 (PMC10618091; doi:10.1038/s41380-023-02117-9)
Supplement: Supplementary file 1 — Supplemental Material [file 41380_2023_2117_MOESM1_ESM.docx]

**Supplemental Figure 1. Manhattan plot of Meta-Analysis GWAS results for DSM-IV PTSD diagnosis. Negative log-transformed *p*-values for SNPs are plotted against base-pair position for each chromosome.**

**
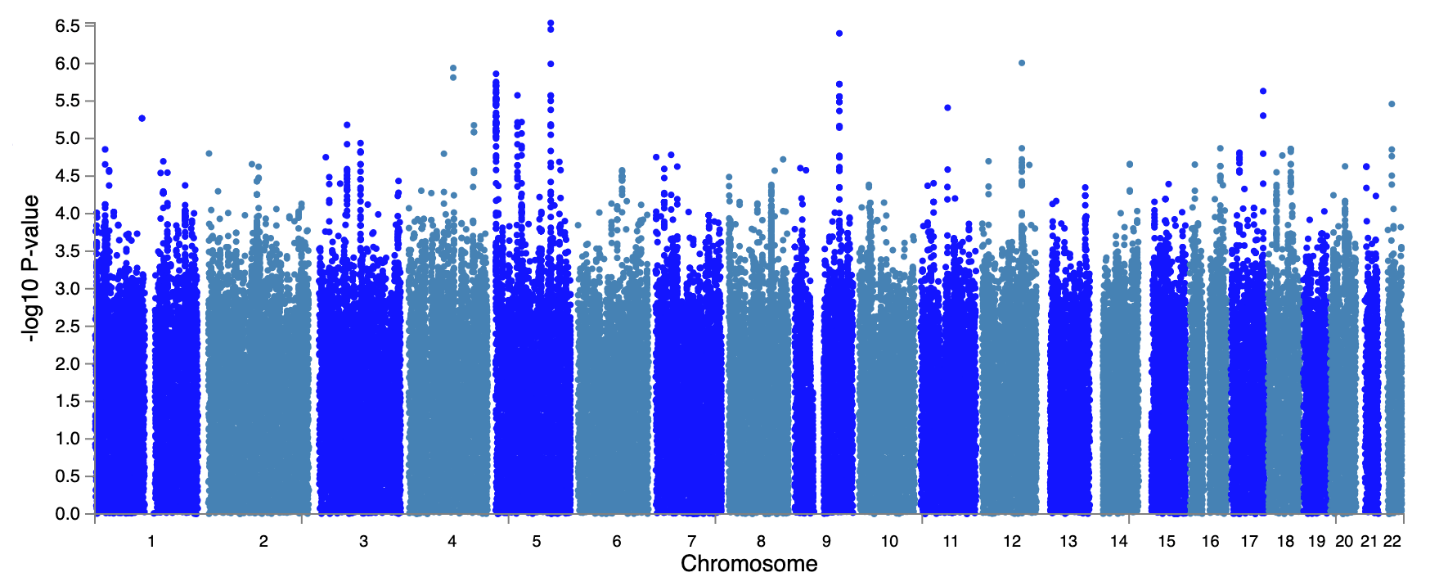
**

**Supplemental Table 1. Descriptive Statistics for GWAS sample in trauma-exposed individuals**

|  | | N | PTSD diagnosis [N(%)] | Age [Mean (SD)] |
| --- | --- | --- | --- | --- |
| Full sample | | 4978 | 363 (7.3) | 32.1 (12.4) |
|  | Male | 2496 | 113 (4.5) | 32.7 (13.3) |
|  | Female | 2482 | 250 (10.1) | 31.5 (11.4) |
| EA | | 3254 | 256 (7.9) | 33.1 (13.0) |
|  | Male | 1770 | 74 (4.2) | 34.0 (14.1) |
|  | Female | 1740 | 182 (10.5) | 32.3 (11.8) |
| AA | | 1468 | 107 (7.3) | 29.6 (10.3) |
|  | Male | 726 | 39 (5.4) | 29.6 (10.6) |
|  | Female | 742 | 68 (9.2) | 29.7 (10.0) |

**Supplemental Table 2. Descriptive statistics for PGC PTSD Polygenic Risk Score (PRS) analyses in individuals of European Ancestry**

|  | | **Full sample (N=3533)** | **Male (N= 1773)** | **Female (N=1760)** |
| --- | --- | --- | --- | --- |
| Age [Mean (SD)] | | 33.2 (13.0) | 34.1 (14.1) | 32.3 (11.8) |
| Family history of alcohol dependence [N(%)] | | 1799 (50.9) | 892 (50.3) | 907 (51.5) |
| DSM-IV Diagnoses | | | | |
|  | PTSD [N(%)] | 261 (7.3) | 75 (4.2) | 186 (10.5) |
|  | Alcohol dependence [N(%)] | 1157 (32.7) | 705 (39.7) | 452 (25.6) |
|  | Cannabis Dependence [N(%)] | 723 (20.4) | 460 (25.9) | 263 (14.9) |
|  | Cocaine Dependence[N(%)] | 363 (10.2) | 215 (12.1) | 148 (8.4) |
|  | Opioid Dependence [N(%)] | 210 (5.9) | 124 (6.9) | 86 (4.8) |

**Supplemental Table 3. Descriptive statistics for MVP PTSD PRS analyses**

|  | | **Full sample** | | | **EA** | | | **AA** | | |
| --- | --- | --- | --- | --- | --- | --- | --- | --- | --- | --- |
|  | | **All (N=5200)** | **Male**  **(N=2595)** | **Female**  **(N=2605)** | **All**  **(N=3522)** | **Male**  **(N=1762)** | **Female**  **(N=1760)** | **All**  **(N=1678)** | **Male**  **(N=833)** | **Female**  **(N=845)** |
| Age [Mean (SD)] | | 32.0(12.3) | 32.6(31.4) | 31.4(11.3) | 33.2(13.0) | 34.1(14.1) | 32.3(11.8) | 29.5(10.3) | 29.4(10.6) | 29.6(9.9) |
| Family history of alcohol dependence [N(%)] | | 2601  (50.0) | 1296  (49.9) | 1305  (50.0) | 1795  (50.9) | 888  (50.3) | 907  (51.5) | 806  (48.0) | 408  (48.9) | 398  (47.1) |
| DSM-IV Diagnoses [N(%)] | |  | | |  | | |  | | |
|  | PTSD | 385 (7.4) | 118 (4.5) | 267 (10.2) | 261 (7.4) | 75 (4.2) | 186 (10.5) | 124 (7.3) | 43 (5.1) | 81 (9.5) |
|  | Alcohol dependence | 1540 (29.6) | 946 (36.4) | 594 (22.8) | 1157 (32.8) | 703 (39.8) | 454 (25.7) | 383 (22.8) | 243 (29.1) | 140 (16.5) |
|  | Cannabis Dependence | 1148 (22.0) | 734 (28.2) | 414 (15.8) | 723 (20.5) | 459 (26.0) | 264 (15.0) | 425 (25.3) | 275 (33.1) | 150 (17.7) |
|  | Cocaine Dependence | 555 (10.6) | 332 (12.7) | 223 (8.5) | 364 (10.3) | 215 (12.2) | 149 (8.4) | 191 (11.3) | 117 (14.0) | 74 (8.7) |
|  | Opioid Dependence | 264 (5.0) | 161 (6.2) | 103 (3.9) | 209 (5.9) | 123 (6.9) | 86 (4.8) | 55 (3.2) | 38 (4.5) | 17 (2.0) |

**Supplemental Table 4. Correlations between outcome variables in PGC-PTSD PRS analyses.**

|  | **PTSD** | **Alcohol dependence** | **Cannabis dependence** | **Cocaine dependence** | **Opioid dependence** |
| --- | --- | --- | --- | --- | --- |
| **PTSD** | 1.000 | 0.130* | 0.078* | 0.062* | 0.077* |
| **Alcohol dependence** | - | 1.000 | 0.255* | 0.323* | 0.186* |
| **Cannabis dependence** | - | - | 1.000 | 0.271* | 0.194* |
| **Cocaine dependence** | - | - | - | 1.000 | 0.236* |

Note: Correlations represent Pearson Correlation coefficients. *p<0.001

**Supplemental Table 5. Correlations between outcome variables in MVP-PTSD PRS analyses.**

|  | EA | | | | | AA | | | | |
| --- | --- | --- | --- | --- | --- | --- | --- | --- | --- | --- |
|  | PTSD | Alcohol dependence | Cannabis dependence | Cocaine dependence | Opioid dependence | PTSD | Alcohol dependence | Cannabis dependence | Cocaine dependence | Opioid dependence |
| PTSD | 1.000 | 0.130** | 0.103** | 0.071** | 0.094** | 1.000 | 0.080* | 0.082** | 0.072* | 0.077* |
| Alcohol dependence | - | 1.000 | 0.333** | 0.349** | 0.226** | - | 1.000 | 0.300** | 0.468** | 0.203** |
| Cannabis dependence | - | - | 1.000 | 0.348** | 0.268** | - | - | 1.000 | 0.205** | 0.108** |
| Cocaine dependence | - | - | - | 1.000 | 0.317** | - | - | - | 1.000 | 0.324** |

Note: Correlations represent Pearson Correlation coefficients. *p<0.01, **p<0.001

**Supplemental Table 6. Meta-analyses of European and African ancestry GWAS. Top 10 SNPs in the overall (N=4989) analyses are reported.**

| **Chr** | **Position** | **SNP** | **Effect Allele** | **Beta** | **p-value** |
| --- | --- | --- | --- | --- | --- |
| 5 | 133179080 | rs2457174 | T | -5.131 | 2.881x10^-7^ |
| 5 | 133178788 | rs2431560 | A | -5.093 | 3.516x10^-7^ |
| 9 | 108213405 | rs9969773 | T | -5.071 | 3.966x10^-7^ |
| 12 | 97269622 | rs71439002 | C | 4.895 | 9.821x10^-7^ |
| 5 | 133210013 | rs2452852 | A | -4.889 | 1.013x10^-6^ |
| 4 | 107339050 | rs7668166 | C | -4.865 | 1.147x10^-6^ |
| 5 | 936899 | rs67697853 | T | 4.829 | 1.372x10^-6^ |
| 4 | 107340838 | rs4443360 | C | -4.806 | 1.538x10^-6^ |
| 5 | 951358 | rs7704095 | T | 4.779 | 1.760x10^-6^ |
| 5 | 946106 | rs56105322 | A | 4.772 | 1.822x10^-6^ |

**Supplemental Table 7. Main and interaction effects of PGC-PTSD PRS and sex on DSM-IV PTSD and substance dependence in COGA.**

| **Outcome Variables** | **Explanatory Variables** | **Estimate(S.E.)** | **OR[CI95%]** | ***p*-value** | ***adjusted p-value*** |
| --- | --- | --- | --- | --- | --- |
| PTSD | **PRS** | **0.121(0.035)** | **1.128[1.065-1.196]** | **0.001**** | **0.007**** |
|  | **Sex** | **0.228(0.031)** | **1.256[1.124-1.320]** | **<0.001***** | **<0.001***** |
|  | PRS*Sex | 0.010(0.036) | 1.010[0.951-1.072] | 0.782 | 1.000 |
| Alcohol dependence | **PRS** | **0.107(0.026)** | **1.112[1.066-1.161]** | **<0.001***** | **<0.001***** |
|  | **Sex** | **-0.178(0.020)** | **0.836[0.809-0.864]** | **<0.001***** | **<0.001***** |
|  | PRS*Sex | -0.004(0.022) | 0.996[0.960-1.032] | 0.851 | 1.000 |
| Cannabis dependence | PRS | 0.063(0.027) | 1.065[1.018-1.114] | 0.021* | 0.105 |
|  | **Sex** | **-0.203(0.024)** | **0.816[0.785-0.849]** | **<0.001***** | **<0.001***** |
|  | PRS*Sex | 0.017(0.027) | 1.017[0.973-1.062] | 0.536 | 1.000 |
| Cocaine dependence | **PRS** | **0.079(0.030)** | **1.082[1.030-1.137]** | **0.008**** | **0.045*** |
|  | **Sex** | **-0.094(0.027)** | **0.910[0.870-0.951]** | **<0.001***** | **<0.001***** |
|  | PRS*Sex | 0.004(0.027) | 1.004[0.960-1.049] | 0.894 | 1.000 |
| Opioid dependence | PRS | 0.063(0.037) | 1.065[1.001-1.132] | 0.093 | 0.380 |
|  | **Sex** | **-0.090(0.032)** | **0.913[0.869-0.963]** | **0.005**** | **0.032*** |
|  | PRS*Sex | 0.023(0.035) | 1.023[0.966-1.084] | 0.505 | 1.000 |

**Note: Bolded are significant associations that remained significant after adjusting p-values for multiple testing using the Benjamini-Hochberg procedure.** Age, principal components and genotype array, and cross-term covariate interactions were included as covariates. All variables were modeled simultaneously.

*p<0.05; **p<0.01; ***p<0.001.

**Supplemental Table 8. Main and interaction effects of PGC-PTSD PRS, Problematic Alcohol Use PRS (Alc PRS), and sex on DSM-IV PTSD and substance dependence in COGA.**

| **Outcome Variables** | **Explanatory Variables** | **Estimate(S.E.)** | **OR[CI95%]** | ***p*-value** | ***adjusted p-value*** |
| --- | --- | --- | --- | --- | --- |
| PTSD | **PTSD PRS** | **0.107 (0.036)** | **1.113[0.048-0.165]** | **0.003**** | **0.009**** |
|  | **Alc PRS** | **0.078 (0.031)** | **1.081[0.027-0.129]** | **0.012*** | **0.030*** |
|  | **Sex** | **0.230 (0.031)** | **1.258[0.179-0.290]** | **<0.001***** | **<0.001***** |
|  | PTSD PRS*Alc PRS | 0.064 (0.031) | 1.066[0.012-0.115] | 0.042* | 0.096 |
|  | PTSD PRS*Sex | 0.002 (0.036) | 1.002[-0.058-0.061] | 0.967 | 1.000 |
|  | Alc PRS*Sex | 0.042 (0.030) | 1.042[-0.009-0.092] | 0.173 | 0.273 |
| Alcohol dependence | **PTSD PRS** | **0.088 (0.026)** | **1.091[0.045-0.132]** | **0.001**** | **0.004**** |
|  | **Alc PRS** | **0.148 (0.021)** | **1.159[0.113-0.183]** | **<0.001***** | **<0.001***** |
|  | **Sex** | **-0.179 (0.020)** | **0.836[-0.211- -0.146]** | **<0.001***** | **<0.001***** |
|  | PTSD PRS*Alc PRS | 0.042 (0.024) | 1.042[0.003-0.081] | 0.074 | 0.148 |
|  | PTSD PRS*Sex | -0.006 (0.022) | 0.994[-0.043-0.030] | 0.780 | 0.900 |
|  | Alc PRS*Sex | -0.037 (0.022) | 0.963[-0.074- -0.001] | 0.095 | 0.167 |
| Cannabis dependence | PTSD PRS | 0.050 (0.027) | 1.051[0.006-0.095] | 0.064 | 0.137 |
|  | **Alc PRS** | **0.084 (0.026)** | **1.087[0.042-0.127]** | **0.001**** | **0.003**** |
|  | **Sex** | **-0.208 (0.024)** | **0.812[-0.247- -0.168]** | **<0.001***** | **<0.001***** |
|  | PTSD PRS*Alc PRS | 0.001 (0.024) | 1.001[-0.039-0.041] | 0.970 | 0.970 |
|  | PTSD PRS*Sex | 0.007 (0.027) | 1.007[-0.037-0.050] | 0.801 | 0.890 |
|  | Alc PRS*Sex | -0.015 (0.025) | 0.985[-0.055-0.026] | 0.549 | 0.748 |
| Cocaine dependence | PTSD PRS | 0.053 (0.031) | 1.054[0.003-0.104] | 0.079 | 0.148 |
|  | **Alc PRS** | **0.136 (0.032)** | **1.145[0.082-0.189]** | **<0.001***** | **<0.001***** |
|  | **Sex** | **-0.091 (0.027)** | **0.913[-0.135- -0.047]** | **0.001**** | **0.003**** |
|  | PTSD PRS*Alc PRS | 0.008 (0.027) | 1.008[-0.037-0.053] | 0.775 | 0.930 |
|  | PTSD PRS*Sex | -0.010 (0.027) | 0.990[-0.055-0.035] | 0.718 | 0.897 |
|  | Alc PRS*Sex | -0.004 (0.029) | 0.996[-0.052-0.044] | 0.889 | 0.952 |
| Opioid dependence | PTSD PRS | 0.053 (0.038) | 1.054[-0.009-0.115] | 0.158 | 0.263 |
|  | **Alc PRS** | **0.124 (0.036)** | **1.132[0.066-0.183]** | **<0.001***** | **<0.001***** |
|  | **Sex** | **-0.084 (0.032)** | **0.919[-0.136- -0.032]** | **0.008**** | **0.021*** |
|  | PTSD PRS*Alc PRS | 0.021 (0.036) | 1.021[-0.038-0.081] | 0.558 | 0.727 |
|  | PTSD PRS*Sex | 0.025 (0.035) | 1.025[-0.032-0.083] | 0.470 | 0.671 |
|  | Alc PRS*Sex | -0.036 (0.035) | 0.964[-0.093-0.021] | 0.299 | 0.448 |

**Note: Bolded are significant associations that remained significant after adjusting p-values for multiple testing using the Benjamini-Hochberg procedure.** Age, principal components and genotype array, and cross-term covariate interactions were included as covariates. All variables were modeled simultaneously.

*p<0.05; **p<0.01; ***p<0.001.

**Supplemental Table 9. Main and interaction effects of PGC-PTSD PRS and DSM-IV alcohol dependence diagnosis on DSM-IV PTSD and substance dependence in COGA.**

| **Outcome Variables** | **Explanatory Variables** | **Beta (S.E.)** | **OR[CI95%]** | **p-value** | ***adjusted p-value*** |
| --- | --- | --- | --- | --- | --- |
| PTSD | | | | |  |
|  | **PRS** | **0.094(0.035)** | **1.098[1.037-1.164]** | **0.007**** | **0.042*** |
|  | **Alcohol dependence** | **0.253(0.031)** | **1.287[1.220-1.357]** | **<0.001***** | **<0.001***** |
|  | **PRS*alcohol dependence** | **0.100(0.034)** | **1.105[1.043-1.168]** | **0.004**** | **0.028*** |
| Cannabis dependence | | | | |  |
|  | PRS | 0.025(0.025) | 1.025[0.985-1.068] | 0.306 | 0.688 |
|  | **Alcohol dependence** | **0.442(0.020)** | **1.555[1.505-1.609]** | **<0.001***** | **<0.001***** |
|  | PRS*alcohol dependence | 0.031(0.022) | 1.031[0.994-1.070] | 0.163 | 0.489 |
| Cocaine dependence | | | | |  |
|  | PRS | 0.037(0.028) | 1.037[0.992-1.085] | 0.181 | 0.465 |
|  | **Alcohol dependence** | **0.481(0.023)** | **1.617[1.557-1.678]** | **<0.001***** | **<0.001***** |
|  | PRS*alcohol dependence | 0.052(0.022) | 1.053[1.016-1.090] | 0.017* | 0.087 |
| Opioid dependence | | | | |  |
|  | PRS | 0.027(0.036) | 1.027[0.968-1.089] | 0.449 | 0.850 |
|  | **Alcohol dependence** | **0.403(0.030)** | **1.537[1.423-1.573]** | **<0.001***** | **<0.001***** |
|  | PRS*alcohol dependence | 0.045(0.031) | 1.046[0.994-1.100] | 0.148 | 0.484 |

**Note: Bolded are significant associations that remained significant after adjusting p-values for multiple testing using the Benjamini-Hochberg procedure.** Sex, age, principal components, genotype array, and cross-term covariate interactions were included as covariates. All variables were modeled simultaneously.

*p<0.05; **p<0.01; ***p<0.001.

**Supplemental Table 10. Main and interaction effects of PGC-PTSD PRS, Problematic Alcohol Use PRS (Alc PRS) and DSM-IV alcohol dependence diagnosis on DSM-IV PTSD and substance dependence in COGA.**

| **Outcome Variables** | **Explanatory Variables** | **Beta (S.E.)** | **OR[CI95%]** | **p-value** | ***adjusted p-value*** |
| --- | --- | --- | --- | --- | --- |
| PTSD | | | | |  |
|  | PTSD PRS | 0.086(0.035) | 1.090[0.028-0.144] | 0.014* | 0.056 |
|  | Alc PRS | 0.050(0.031) | 1.051[-0.001-0.101] | 0.109 | 0.290 |
|  | **Alcohol dependence** | **0.248(0.033)** | **1.281[0.193-0.302]** | **<0.001***** | **<0.001***** |
|  | PTSD PRS*Alc PRS | 0.046(0.030) | 1.047[-0.004-0.009] | 0.130 | 0.283 |
|  | **PTSD PRS*alcohol dependence** | **0.100(0.035)** | **1.105[0.041-0.155]** | **0.005**** | **0.024*** |
|  | Alc PRS*alcohol dependence | -0.034(0.030) | 0.967[-0.084-0.015] | 0.256 | 0.409 |
| Cannabis dependence | | | | |  |
|  | PTSD PRS | 0.020(0.024) | 1.020[-0.021-0.060] | 0.419 | 0.558 |
|  | Alc PRS | 0.039(0.025) | 1.040[-0.001-0.080] | 0.109 | 0.261 |
|  | **Alcohol dependence** | **0.426(0.019)** | **1.531[0.395-0.457]** | **<0.001***** | **<0.001***** |
|  | PTSD PRS*Alc PRS | -0.020(0.021) | 0.980[-0.057-0.016] | 0.358 | 0.505 |
|  | PTSD PRS*alcohol dependence | 0.023(0.023) | 1.023[-0.014-0.060] | 0.314 | 0.471 |
|  | Alc PRS*alcohol dependence | 0.001(0.020) | 1.001[-0.033-0.034] | 0.974 | 1.016 |
| Cocaine dependence | | | | |  |
|  | PTSD PRS | 0.018(0.028) | 1.018[-0.027-0.064] | 0.514 | 0.616 |
|  | **Alc PRS** | **0.078(0.032)** | **1.081[0.026-0.131]** | **0.014*** | **0.048*** |
|  | **Alcohol dependence** | **0.475(0.024)** | **1.608[0.436-0.514]** | **<0.001***** | **<0.001***** |
|  | PTSD PRS*Alc PRS | -0.015(0.025) | 0.985[-0.059-0.027] | 0.538 | 0.614 |
|  | PTSD PRS*alcohol dependence | 0.033(0.022) | 1.034[0.003-0.067] | 0.131 | 0..262 |
|  | Alc PRS*alcohol dependence | 0.032(0.022) | 1.033[-0.005-0.068] | 0.153 | 0.282 |
| Opioid dependence | | | | |  |
|  | PTSD PRS | 0.025(0.036) | 1.025[-0.035-0.084] | 0.496 | 0.626 |
|  | Alc PRS | 0.079(0.035) | 1.082[0.021-0.138] | 0.025* | 0.075 |
|  | **Alcohol dependence** | **0.391(0.029)** | **1.478[0.344-0.439]** | **<0.001***** | **<0.001***** |
|  | PTSD PRS*Alc PRS | 0.001(0.035) | 1.001[-0.059-0.061] | 0.977 | 0.977 |
|  | PTSD PRS*alcohol dependence | 0.042(0.032) | 1.043[-0.010-0.093] | 0.187 | 0.320 |
|  | Alc PRS*alcohol dependence | -0.007(0.025) | 0.993[-0.048-0.034] | 0.774 | 0.811 |

**Note: Bolded are significant associations that remained significant after adjusting p-values for multiple testing using the Benjamini-Hochberg procedure.** Sex, age, principal components, genotype array, and cross-term covariate interactions were included as covariates. All variables were modeled simultaneously.*p<0.05; **p<0.01; ***p<0.001.

**Supplemental Table 11. Main and interaction effects of MVP-PTSD PRS and sex on DSM-IV PTSD and substance dependence in COGA stratified by EA and AA ancestry.**

|  | | **EA** | | | | **AA** | | | |
| --- | --- | --- | --- | --- | --- | --- | --- | --- | --- |
| **Outcome variables** | **Explanatory variables** | **Beta(S.E.)** | **OR [CI95%]** | **p-value** | **Adjusted p-value** | **Beta(S.E.)** | **OR [CI95%]** | **p-value** | **Adjusted p-value** |
| PTSD | |  |  |  |  |  |  |  |  |
|  | PRS | 0.069(0.038) | 1.071[1.006-1.141] | 0.070 | 0.315 | 0.010(0.162) | 1.010[0.774-1.317] | 0.950 | 1.000 |
|  | Sex | 0.315(0.232) | 1.370[0.935-2.007] | 0.174 | 0.559 | 0.130(2.450) | 1.138[0.020-64.071] | 0.958 | 1.000 |
|  | PRS*sex | 0.086(0.240) | 1.089[0.734-1.619] | 0.719 | 1.000 | 0.033(2.667) | 1.033[0.012-83.179] | 0.975 | 1.000 |
| Alcohol dependence | |  |  |  |  |  |  |  |  |
|  | PRS | 0.025(0.026) | 1.025[0.982-1.071] | 0.332 | 0.933 | 0.087(0.140) | 1.090[0.866-1.374] | 0.533 | 1.000 |
|  | Sex | -0.102(0.179) | 0.903[0.673-1.210] | 0.567 | 1.000 | -0.243(2.692) | 0.784[0.009-65.071] | 0.928 | 1.000 |
|  | PRS*sex | 0.076(0.182) | 1.078[0.800-1.456] | 0.674 | 1.000 | 0.031(2.926) | 1.031[0.008-127.103] | 0.991 | 1.000 |
| Cannabis dependence | |  |  |  |  |  |  |  |  |
|  | PRS | 0.044(0.029) | 1.044[0.997-1.095] | 0.126 | 0.472 | 0.053(0.166) | 1.054[0.801-1.385] | 0.751 | 1.000 |
|  | Sex | -0.163(0.202) | 0.849[0.608-1.185] | 0.421 | 1.000 | -0.240(2.927) | 0.786[0.006-96.931] | 0.935 | 1.000 |
|  | PRS*sex | 0.039(0.204) | 1.039[0.743-1.456] | 0.848 | 1.000 | 0.005(3.185) | 1.006[0.005-189.615] | 0.999 | 1.000 |
| Cocaine dependence | |  |  |  |  |  |  |  |  |
|  | PRS | 0.047(0.032) | 1.048[0.993-1.105] | 0.149 | 0.515 | 0.015(0.156) | 1.015[0.785-1.312] | 0.924 | 1.000 |
|  | Sex | 0.058(0.136) | 1.059[0.847-1.327] | 0.670 | 1.000 | -0.133(1.977) | 0.875[0.033-22.601] | 0.946 | 1.000 |
|  | PRS*sex | 0.158(0.137) | 1.171[0.935-1.468] | 0.248 | 0.744 | -0.007(2.146) | 0.993[0.029-33.919] | 0.998 | 1.000 |
| Opioid dependence | |  |  |  |  |  |  |  |  |
|  | PRS | 0.010(0.040) | 1.010[0.946-1.077] | 0.799 | 1.000 | -0.004(0.186) | 0.996[0.734-1.352] | 0.983 | 1.000 |
|  | Sex | -0.039(0.153) | 0.961[0.747-1.237] | 0.798 | 1.000 | -0.099(3.210) | 0.905[0.004-177.860] | 0.975 | 1.000 |
|  | PRS*sex | 0.052(0.154) | 1.053[0.817-1.357] | 0.737 | 1.000 | -0.084(3.494) | 0.919[0.002-288.011] | 0.981 | 1.000 |

**Note:** Age, principal components, genotype array, and cross-term covariate interactions were included as covariates. All variables were modeled simultaneously. Adjusted p-values were calculated using the Benjamini-Hochberg procedure.

**Supplemental Table 12. Main and interaction effects of MVP-PTSD PRS, Problematic Alcohol Use PRS (Alc PRS), and sex on DSM-IV PTSD and substance dependence in COGA stratified by EA and AA ancestry.**

|  | | **EA** | | | | **AA** | | | |
| --- | --- | --- | --- | --- | --- | --- | --- | --- | --- |
| **Outcome variables** | **Explanatory variables** | **Beta(S.E.)** | **OR [CI95%]** | **p-value** | **Adjusted p-value** | **Beta(S.E.)** | **OR [CI95%]** | **p-value** | **Adjusted p-value** |
| PTSD | |  |  |  |  |  |  |  |  |
|  | PTSD PRS | 0.063(0.038) | 1.065[0.000-0.126] | 0.100 | 1.000 | 0.153(0.187) | 1.165[-0.156-0.461] | 0.415 | 1.000 |
|  | Alc PRS | 0.091(0.144) | 1.095[-0.146-0.327] | 0.528 | 1.000 | -0.127(0.779) | 0.881[-1.409-1.155] | 0.870 | 1.000 |
|  | Sex | 0.299(0.233) | 1.349[-0.084-0.682] | 0.199 | 1.000 | 0.152(1.576) | 1.164[-2.440-2.745] | 0.923 | 1.000 |
|  | PTSD PRS *Alc PRS | 0.010(0.149) | 1.010[-0.236-0.256] | 0.946 | 1.000 | -0.078(0.808) | 0.925[-1.408-1.251] | 0.923 | 1.000 |
|  | PTSD PRS*sex | 0.069(0.241) | 1.071[-0.328-0.465] | 0.776 | 1.000 | -0.004(1.721) | 0.996[-2.834-2.827] | 0.998 | 0.998 |
|  | Alc PRS*sex | 0.037(0.030) | 1.038[-0.013-0.086] | 0.226 | 1.000 | -0.047(0.061) | 0.954[-0.147-0.053] | 0.439 | 1.000 |
| Alcohol dependence | |  |  |  |  |  |  |  |  |
|  | PTSD PRS | 0.024(0.026) | 1.024[-0.018-0.067] | 0.342 | 1.000 | 0.231(0.143) | 1.260[-0.004-0.467] | 0.107 | 1.000 |
|  | Alc PRS | 0.177(0.104) | 1.194[0.006-0.349] | 0.089 | 1.000 | -0.042(0.544) | 0.959[-0.936-0.852] | 0.938 | 1.000 |
|  | Sex | -0.121(0.179) | 0.886[-0.416-0.174] | 0.500 | 1.000 | -0.157(1.443) | 0.566[-2.530-2.217] | 0.914 | 1.000 |
|  | PTSD PRS *Alc PRS | 0.027(0.105) | 1.027[-0.145-0.199] | 0.798 | 1.000 | 0.030(0.541) | 1.030[-0.860-0.920] | 0.956 | 1.000 |
|  | PTSD PRS*sex | 0.056(0.183) | 1.058[-0.245-0.358] | 0.758 | 1.000 | -0.061(1.571) | 0.941[-2.645-2.524] | 0.969 | 1.000 |
|  | Alc PRS*sex | -0.038(0.023) | 0.963[-0.076- -0.001] | 0.091 | 1.000 | -0.023(0.047) | 0.977[-0.100-0.054] | 0.620 | 1.000 |
| Cannabis dependence | |  |  |  |  |  |  |  |  |
|  | PTSD PRS | 0.042(0.029) | 1.043[-0.005-0.089] | 0.145 | 1.000 | 0.168(0.186) | 1.183[-0.139-0.474] | 0.368 | 1.000 |
|  | Alc PRS | 0.175(0.107) | 1.191[-0.001-0.350] | 0.102 | 1.000 | -0.167(0.406) | 0.846[-0.836-0.501] | 0.680 | 1.000 |
|  | Sex | -0.177(0.204) | 0.838[-0.511-0.158] | 0.386 | 1.000 | -0.174(1.609) | 0.840[-2.821-2.472] | 0.914 | 1.000 |
|  | PTSD PRS *Alc PRS | 0.088(0.109) | 1.092[-0.092-0.268] | 0.421 | 1.000 | 0.173(0.417) | 1.189[-0.513-0.858] | 0.678 | 1.000 |
|  | PTSD PRS*sex | 0.026(0.206) | 1.026[-0.313-0.365] | 0.899 | 1.000 | -0.068(1.755) | 0.934[-2.955-2.820] | 0.969 | 1.000 |
|  | Alc PRS*sex | -0.013(0.025) | 0.987[-0.054-0.028] | 0.601 | 1.000 | -0.065(0.045) | 0.937[-0.140-0.010] | 0.151 | 1.000 |
| Cocaine dependence | |  |  |  |  |  |  |  |  |
|  | PTSD PRS | 0.047(0.032) | 1.048[-0.006-0.100] | 0.147 | 0.980 | 0.015(0.218) | 1.015[-0.344-0.374] | 0.944 | 1.000 |
|  | Alc PRS | 0.227(0.127) | 1.255[0.019-0.227] | 0.073 | 1.000 | 0.032(0.605) | 1.033[-0.964-1.027] | 0.958 | 1.000 |
|  | Sex | 0.036(0.136) | 1.037[-0.188-0.259] | 0.794 | 1.000 | -0.192(1.491) | 0.825[-2.644-2.261] | 0.898 | 1.000 |
|  | PTSD PRS *Alc PRS | 0.092(0.128) | 1.096[-0.119-0.303] | 0.474 | 1.000 | -0.024(0.625) | 0.976[-1.052-1.003] | 0.969 | 1.000 |
|  | PTSD PRS*sex | 0.134(0.137) | 1.143[-0.091-0.359] | 0.327 | 1.000 | 0.060(1.622) | 1.062[-1.148-0.365] | 0.971 | 1.000 |
|  | Alc PRS*sex | -0.017(0.030) | 0.983[-0.066-0.032] | 0.564 | 1.000 | 0.005(0.063) | 1.005[-0.098-0.108] | 0.937 | 1.000 |
| Opioid dependence | |  |  |  |  |  |  |  |  |
|  | PTSD PRS | 0.009(0.040) | 1.009[-0.056-0.075] | 0.816 | 1.000 | 0.011(0.207) | 1.011[-0.329-0.350] | 0.959 | 1.000 |
|  | Alc PRS | 0.129(0.175) | 1.138[-0.159-0.417] | 0.461 | 1.000 | -0.245(2.086) | 0.783[-3.676-3.187] | 0.907 | 1.000 |
|  | Sex | -0.057(0.153) | 0.945[-0.309-0.194] | 0.707 | 1.000 | -0.137(2.034) | 0.872[-3.484-3.209] | 0.946 | 1.000 |
|  | PTSD PRS *Alc PRS | 0.010(0.179) | 1.010[-0.285-0.304] | 0.957 | 1.000 | 0.227(2.180) | 1.255[-3.359-3.813] | 0.917 | 1.000 |
|  | PTSD PRS*sex | 0.033(0.154) | 1.034[-0.221-0.286] | 0.832 | 1.000 | -0.052(2.221) | 0.949[-3.707-3.602] | 0.981 | 0.997 |
|  | Alc PRS*sex | -0.028(0.036) | 0.972[-0.087-0.031] | 0.442 | 1.000 | 0.051(0.079) | 1.052[-0.079-0.182] | 0.517 | 1.000 |

**Note:** Age, principal components, genotype array, and cross-term covariate interactions were included as covariates. All variables were modeled simultaneously. Adjusted p-values were calculated using the Benjamini-Hochberg procedure.

**Supplemental Table 13. Main and interaction effects of MVP-PTSD PRS, Problematic Alcohol Use PRS (Alc PRS), and alcohol dependence on DSM-IV PTSD and substance dependence in COGA stratified by EA and AA ancestry.**

|  | | **EA** | | |  | **AA** | | |  |
| --- | --- | --- | --- | --- | --- | --- | --- | --- | --- |
| **Outcome variables** | **Explanatory variables** | **Beta(S.E.)** | **OR [CI95%]** | **p-value** | **Adjusted p-value** | **Beta(S.E.)** | **OR [CI95%]** | **p-value** | **Adjusted p-value** |
| PTSD | |  |  |  |  |  |  |  |  |
|  | PRS | 0.058(0.038) | 1.059[0.997-1.127] | 0.121 | 0.544 | 0.018(0.203) | 1.018[0.729-1.423] | 0.928 | 1.000 |
|  | Alcohol dependence | 0.219(0.270) | 1.244[0.7977-1.940] | 0.418 | 0.836 | 0.151(2.669) | 1.162[0.014-93.784] | 0.955 | 1.000 |
|  | PRS*alcohol dependence | -0.044(0.280) | 0.956[0.603-1.517] | 0.875 | 1.000 | 0.040(2.926) | 1.040[0.008-128.124] | 0.989 | 0.989 |
| Cannabis dependence | |  |  |  |  |  |  |  |  |
|  | PRS | 0.038(0.026) | 1.038[0.996-1.084] | 0.140 | 0.504 | 0.035(0.197) | 1.035[0.749-1.430] | 0.860 | 1.000 |
|  | Alcohol dependence | 0.391(0.348) | 1.478[0.834-2.622] | 0.260 | 0.624 | 0.333(4.549) | 1.395[0.000-2482.446] | 0.942 | 1.000 |
|  | PRS*alcohol dependence | -0.051(0.360) | 0.950[0.525-1.716] | 0.886 | 1.000 | 0.084(4.977) | 1.087[0.000-2904.948] | 0.987 | 1.000 |
| Cocaine dependence | |  |  |  |  |  |  |  |  |
|  | PRS | 0.042(0.031) | 1.042[0.991-1.097] | 0.178 | 0.492 | 0.020(0.188) | 1.020[0.748-1.390] | 0.915 | 1.000 |
|  | Alcohol dependence | 0.519(0.338) | 1.680[0.962-2.929] | 0.125 | 0.500 | 0.475(3.858) | 1.608[0.002-915.985] | 0.902 | 1.000 |
|  | PRS*alcohol dependence | 0.038(0.349) | 1.038[0.585-1.842] | 0.914 | 1.000 | -0.115(4.213) | 0.891[0.000-911.416] | 0.978 | 1.000 |
| Opioid dependence | |  |  |  |  |  |  |  |  |
|  | PRS | 0.002(0.039) | 1.002[0.940-1.068] | 0.953 | 1.000 | 0.007(0.233) | 1.007[0.686-1.476] | 0.976 | 1.000 |
|  | Alcohol dependence | 0.356(0.355) | 1.427[0.796-2.559] | 0.315 | 0.667 | 0.169(3.710) | 1.184[0.002-529.535] | 0.964 | 1.000 |
|  | PRS*alcohol dependence | -0.047(0.368) | 0.954[0.521-1.748] | 0.899 | 1.000 | 0.096(4.070) | 1.100[0.001-888.913] | 0.981 | 1.000 |

**Note:** Sex, age, principal components, genotype array, and cross-term covariate interactions were included as covariates. All variables were modeled simultaneously. Adjusted p-values were calculated using the Benjamini-Hochberg procedure.

**Supplemental Table 14. Main and interaction effects of MVP-PTSD PRS and alcohol dependence on DSM-IV PTSD and substance dependence in COGA stratified by EA and AA ancestry.**

|  | | **EA** | | |  | **AA** | | |  |
| --- | --- | --- | --- | --- | --- | --- | --- | --- | --- |
| **Outcome variables** | **Explanatory variables** | **Beta(S.E.)** | **OR [CI95%]** | **p-value** | **Adjusted p-value** | **Beta(S.E.)** | **OR [CI95%]** | **p-value** | **Adjusted p-value** |
| PTSD | |  |  |  |  |  |  |  |  |
|  | PTSD PRS | 0.059(0.049) | 1.061[-0.022-0.141] | 0.231 | 0.792 | 0.227(0.553) | 1.255[-0.683-1.137] | 0.410 | 0.855 |
|  | Alc PRS | 0.134(0.154) | 1.143[-0.119-0.387] | 0.384 | 0.877 | 0.294(3.881) | 1.342[-6.090-6.678] | 0.076 | 0.912 |
|  | Alcohol dependence | 0.245(0.187) | 1.278[-0.063-0.554] | 0.190 | 0.701 | 0.380(0.432) | 1.462[-0.330-1.091] | 0.880 | 1.000 |
|  | PTSD PRS *Alc PRS | 0.060(0.158) | 1.062[-0.199-0.319] | 0.704 | 1.000 | -0.468(03.962) | 0.626[-0.212-0.173] | 0.906 | 1.000 |
|  | PTSD PRS*alcohol dependence | -0.009(0.193) | 0.991[-0.327-0.309] | 0.962 | 1.000 | -0.230(0.441) | 0.795[-0.955-0.496] | 0.603 | 0.998 |
|  | Alc PRS*alcohol dependence | -0.039(0.030) | 0.962[-0.088-0.010] | 0.186 | 0.811 | -0.089(0.064) | 0.915[-0.194-0.016] | 0.161 | 0..772 |
| Cannabis dependence | |  |  |  |  |  |  |  |  |
|  | PTSD PRS | 0.048(0.050) | 1.049[-0.034-0.130] | 0.336 | 0.848 | 0.117(0.308) | 1.124[-0.390-0.624] | 0.705 | 1.000 |
|  | Alc PRS | 0.132(0.116) | 1.141[-0.059-0.324] | 0.256 | 0.722 | -0.034(1.915) | 0.967[-3.185-3.117] | 0.986 | .986 |
|  | Alcohol dependence | 0.382(0.237) | 1.465[-0.007-0.771] | 0.106 | 0.636 | 0.321(0.290) | 1.379[-0.155-0.798] | 0.268 | 0.714 |
|  | PTSD PRS*Alc PRS | 0.082(0.118) | 1.085[-0.111-0.276] | 0.485 | 0.895 | 0.060(1.959) | 1.062[-3.162-3.283] | 0.975 | 0.995 |
|  | PTSD PRS*alcohol dependence | -0.058(0.246) | 0.944[-0.464-0.347] | 0.813 | 1.000 | 0.099(0.303) | 1.104[-0.400-0.598] | 0.744 | 1.000 |
|  | Alc PRS*alcohol dependence | -0.016(0.019) | 0.984[-0.048-0.015] | 0.400 | 0.872 | -0.048(0.042) | 0.953[-0.117-0.021] | 0.255 | 0.765 |
| Cocaine dependence | |  |  |  |  |  |  |  |  |
|  | PTSD PRS | 0.005(0.052) | 1.005[-0.080-0.091] | 0.921 | 1.000 | -0.077(0.550) | 0.926[-0.982-0.828] | 0.889 | 1.000 |
|  | Alc PRS | 0.248(0.142) | 1.281[0.014-0.481] | 0.081 | 0.777 | 0.305(3.564) | 1.357[-5.558-6.168] | 0.932 | 1.000 |
|  | Alcohol dependence | 0.689(0.228) | 1.992[0.315-1.064] | 0.002** | 0.096 | 0.690(0.410) | 1.994[0.015-1.366] | 0.093 | 0.744 |
|  | PTSD PRS*Alc PRS | 0.166(0.145) | 1.181[-0.071-0.404] | 0.249 | 0.796 | -0.306(3.649) | 0.736[-6.308-5.696] | 0.933 | 1.000 |
|  | PTSD PRS*alcohol dependence | 0.222(0.237) | 1.249[-0.167-0.611] | 0.348 | 0.835 | -0.304(0.421) | 0.738[-0.996-0.389] | 0.470 | 0.902 |
|  | Alc PRS*alcohol dependence | -0.005(0.018) | 0.995[-0.034-0.024] | 0.774 | 1.000 | 0.034(0.056) | 1.035[-0.057-0.125] | 0.539 | 0.924 |
| Opioid dependence | |  |  |  |  |  |  |  |  |
|  | PTSD PRS | -0.023(0.053) | 0.977[-0.111-0.065] | 0.668 | 1.000 | -0.205(0.653) | 0.815[-1.278-0.869] | 0.754 | 1.000 |
|  | Alc PRS | 0.147(0.197) | 1.158[-0.178-0.471] | 0.457 | 0.914 | 0.337(5.050) | 1.401[-7.969-8.644] | 0.947 | 1.000 |
|  | Alcohol dependence | 0.548(0.230) | 1.730[0.171-0.926] | 0.017* | 0.408 | 0.876(0.367) | 2.401[0.272-1.480] | 0.017* | 0.272 |
|  | PTSD PRS*Alc PRS | 0.056(0.201) | 1.058[-0.275-0.387] | 0.781 | 1.000 | -0.382(5.215) | 0.682[-8.961-8.196] | 0.942 | 1.000 |
|  | PTSD PRS*alcohol dependence | 0.160(0.240) | 1.174[-0.235-0.555] | 0.504 | 0..896 | -0.649(0.394) | 0.523[-1.298-0.000] | 0.100 | 0..685 |
|  | Alc PRS*alcohol dependence | -0.030(0.019) | 0.970[-0.061-0.001] | 0.113 | 0.602 | 0.089(0.067) | 1.093[-0.022-0.199] | 0.186 | 0.744 |

**Note:** Sex, age, principal components, genotype array, and cross-term covariate interactions were included as covariates. All variables were modeled simultaneously. Adjusted p-values were calculated using the Benjamini-Hochberg procedure.
